# The Antimicrobial Efficacy of Topically Applied Mafenide Acetate, Citric Acid and Wound Irrigation Solutions Lavanox and Prontosan against *Pseudomonas aeruginosa*

**DOI:** 10.3390/antibiotics13010042

**Published:** 2024-01-03

**Authors:** Mahsa Bagheri, Andreas Zoric, Maria von Kohout, Paul C. Fuchs, Jennifer Lynn. Schiefer, Christian Opländer

**Affiliations:** 1 Department of Plastic and Aesthetic Surgery, Hand Surgery, HELIOS Hospital Emil von Behring, Walterhoeferstr. 11, 14165 Berlin, Germany; 2 Plastic, Reconstructive and Aesthetic Surgery, RKH Hospital Bietigheim-Vaihingen, Riedstr. 12, 74321 Bietigheim-Bissingen, Germany; 3 Plastic Surgery, Hand Surgery, Burn Center, Cologne-Merheim Hospital, Witten/Herdecke University, Ostmerheimer Str. 200, 51109 Cologne, Germany; 4 Institute for Research in Operative Medicine (IFOM), Cologne-Merheim Medical Center, Witten/Herdecke University, Ostmerheimer Str. 200, 51109 Cologne, Germany

**Keywords:** burns, *Pseudomonas aeruginosa*, wound irrigation solutions, antimicrobial efficacy, ex vivo human skin wound model

## Abstract

Since burn wound infections caused by *Pseudomonas aeruginosa* (PA) lead to major complications and sepsis, this study evaluates the antimicrobial efficacy of the wound irrigation solutions Prontosan (PRT), Lavanox (LAV), citric acid (CA) and mafenide acetate (MA) using microbiology assays and an ex vivo skin wound model. In suspension assays, all the solutions showed significant reductions in bacterial number (log_10_ reduction: CA 5.77; LAV 4.91; PRT 4.74; MA 1.23). The biofilm assay revealed that PRT and LAV reduced biofilm formation by ~25% after a 15 min treatment, while PRT was most effective after a 24 h treatment (~68%). The number of PA in biofilms measured directly after a 15 min treatment was reduced most effectively with CA and LAV (log_10_ reductions ~2.5), whereas after a 24 h treatment, all solutions achieved only 1.36–1.65 log_10_ reductions. In the skin wound model, PRT and LAV provided the highest bacterial reduction after a 15 min treatment (log_10_ reduction 1.8–1.9), while MA was more effective after a 22 h treatment (log_10_ reduction 3.6). The results demonstrated the antimicrobial efficacy of all solutions against PA. Further investigation is needed to explore the potential clinical applications of a combination or alternating use of these solutions for infection prophylaxis and treatment of wound infections caused by PA.

## 1. Introduction

Burn patient care optimization is an ongoing process. Treatment of patients with extensive burn injuries requires specialized care with specialized staff, intensive care units and in-patient care [1].

In burn injuries, a bacterial infection is one of the most potentially severe complications that can occur during treatment [2,3]. With an early onset, burn patients are particularly at risk of developing sepsis, and subsequent death, due to infection because of the immune suppression caused by severe burn injuries [2,4]. Burn wound sepsis is the main cause of mortality after a burn trauma [5]. Initially, the burn wound is sterile, but colonization by the normal skin flora starts within the first 48 h after injury and can lead to wound infection [6]. Burn wounds present an ideal environment for bacteria, due to the necrotic tissue and the protein-rich wound exudate [4]. Infected burn patients have a death rate that is double that of uninfected patients [7].

Typically, most nosocomial infections of burn wounds are caused by multidrug-resistant (MDR) Gram-negative bacteria [8]. *Pseudomonas* spp., especially *Pseudomonas aeruginosa* (PA), is most typically isolated from infected burn wounds [9]. PA is an aerobic, sporeless bacterium having intrinsic resistance mechanisms against antibiotics that can produce several extracellular virulence factors [10,11]. Clinically, PA wounds appear yellow-greenish with a pungent-fruity odour. In patients immunocompromised due to extensive burns, an invasive PA infection can lead to potentially fatal sepsis, often with ecthyma gangraenosum as a complication, which manifests as purple-bluish ‘punched-out lesions´ [12]. Systemically, PA wound infections can be treated with anti-pseudomonal ß-lactam antibiotics, e.g., piperacillin/tazobactam, cephalosporine, fluoroquinolones and carbapenems. The use of systemic antibiotics can perpetuate a vicious cycle of growing antimicrobial resistance and increasing MDR bacteria, which already have a high occurrence in burn centres [13].

PA infection is particularly persistent due to its biofilm production triggered by the burn wound exudate, which significantly prolongs healing time [6]. A biofilm is a highly organized exopolysaccharide matrix that encloses bacterial cells. This matrix provides an efficient barrier that restricts the penetration of chemically reactive biocides, cationic antibiotics and antimicrobial peptides [14,15,16]. Due to the resistance and biofilm production of PA, surgical excision via aggressive debridement of necrotic lesions is often required to treat infected wound areas [5,17].

In addition to systemic antibiotics, infected burn wounds can be treated topically. Several classes of topical antibiotics are currently available on the market. Commonly used antibiotic topical agents are mafenide acetate (MA) and citric acid (CA) [18,19]. MA is a sulphonamide-type antibiotic MA is not only a highly effective broad-spectrum antibiotic but is also toxic to cells and can delay wound healing. In addition, MA is enzymatically converted into a carbonic anhydrase inhibitor (p-sulfamylyanic acid), which can cause metabolic acidosis [20,21,22]. CA is an efficient therapy at a dosage of 3%, and studies have shown that CA can stimulate the production of granulation tissues after seven applications and has no toxic effects on fibroblasts [19,23]. 

Furthermore, different wound irrigation solutions can be used for cleansing the wound and as wound dressings. Lavanox (LAV) is a wound irrigation solution of the group of oxidative halides that contains an electrochemically activated mineral salt solution containing 0.08% sodium hypochlorite (NaOCl) [24]. Historically, sodium hypochlorite was in use due to its broad antibacterial spectrum and effectiveness at dissolving biofilms [25,26,27]. Nevertheless, in vitro studies have shown that Lavanox can induce cell toxicity [28].

Prontosan (PRT) is a wound irrigation solution that contains purified water, polyhexanide (0.1%), and betadine (0.1%) [29]. Polyhexanide, a synthetic molecule, achieves its disinfecting effect in a similar way to antimicrobial peptides by binding to the cell walls of bacteria and damaging them by breaking open the lipopolysaccharide layer [30]. Betadine is a moderate surfactant that repels water, dirt and debris and clears bacteria and biofilm from a wound [31]. Furthermore, betaine also promotes the removal of protein coatings on wounds and interrupts biofilm cell-to-cell communication via homoserine lactone. The solution is widely used to clean wounds and moisten and lubricate absorbent wound dressings for ulcers, burns, post-surgical wounds and abrasions [32].

Although all four solutions are frequently used for the treatment/prophylaxis of bacterial wound infections, there is a lack of studies comparing their antibacterial efficacies, in particular against PA, under realistic clinical conditions. Since quantification of the anti-bacterial properties in this context is important, we have established in preliminary studies a quantifiable human skin wound contamination model which simulates clinical reality more closely than standard assays [33].

Therefore, using this human skin model and other microbiology assays, we evaluated and compared the antimicrobial properties of MA, CA, PRT or LAV against PA. 

## 2. Results

### 2.1. Antimicrobial Efficacy of Antiseptics/Wound Irrigation Solutions Using In Vitro Assays

**Figure 1:**
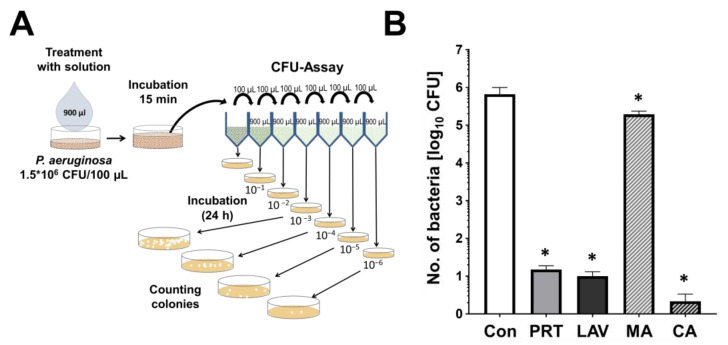
The antimicrobial efficacy against planktonic *P. aeruginosa. (***A**) Suspension method and consequent CFU assay to determine the antimicrobial effects of solutions on bacteria (*Pseudomonas aeruginosa*, PA). (**B**) The obtained number of PA after a 15 min treatment with 0.9% NaCl (control, Con), Prontosan (PRT), Lavanox (LAV), 5% mafenide acetate (MA) or 2% citric acid (CA) (*n* = 5, * *p* ≤ 0.05).

The treatment of planktonic PA with wound irrigation solutions showed significant reductions in log_10_ CFU after 15 min of treatment for all solutions compared to the control, as shown in Figure 1B. The highest log_10_ CFU reduction (5.77) was obtained after the treatment with CA, followed by LAV (4.91), Prontosan (4.74) and MA (1.23). Thus, after the treatment with MA for 15 min, the reduction was the lowest compared to LAV, PRT or CA. All treatments revealed a significant log_10_ CFU reduction and thus antimicrobial effects. 

**Figure 2:**
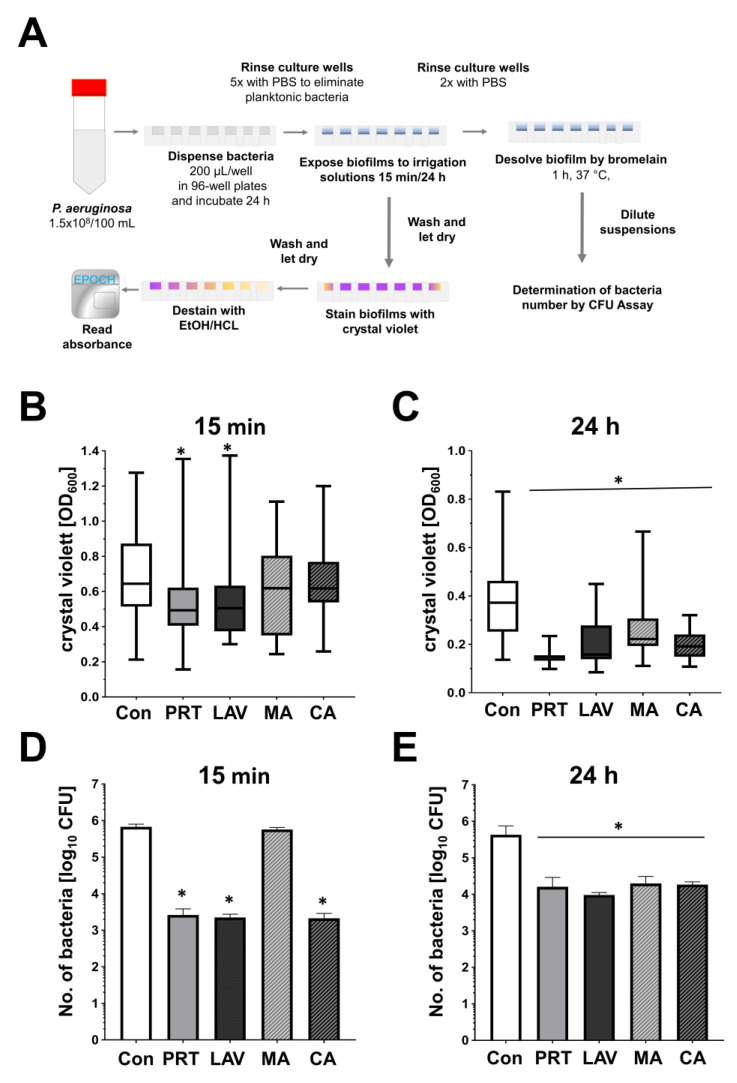
Effects of Prontosan, Lavanox, mafenide acetate and citric acid on *P. aeruginosa* biofilm formation. (**A**) The workflow of biofilm experiments. Box blots with whiskers display the minimum, maximum, median, upper and lower quartiles of obtained values of biofilm mass stained with crystal violet after (**B**) 15 min or (**C**) 24 h treatments with 0.9% NaCl (Con), Prontosan (PRT), Lavanox (LAV), mafenide acetate (MA) or 2% citric acid (CA). The numbers of bacteria (mean + SD) found in biofilms are shown in (**D**) after 15 min or (**E**) 24 h of treatment (*n* = 5, * *p* ≤ 0.05).

In the biofilm assay, we found reductions in bacterial biofilm formation after 15 min of treatment (Figure 2B). Compared to the control (0.69 ± 0.25), the greatest reductions in absorbance/biofilm formation were achieved with PRT (0.52 ± 0.21) and LAV (0.52 ± 0.21), followed by MA (0.59 ± 0.24) and CA (0.64 ± 0.25). After a 24 h treatment (Figure 2C), a significant reduction in biofilm formation was achieved with PRT (0.14 ± 0.02), followed by CA (0.19 ± 0.05), LAV (0.20 ± 0.08) and MA (0.21 ± 0.11) compared to the control using NaCl (0.36 ± 0.30).

In addition, bacteria of the biofilm were quantified by using bromelain digestion of the biofilm. After 15 min of treatment, all solutions except MA showed relevant reductions in bacterial number in biofilms, as shown in Figure 2D. Here, the calculated log_10_ CFU reduction for MA was with a value of 0.07 low, whereas significant log_10_ CFU reductions were achieved by CA (2.50), followed by LAV (2.48) and PRT (2.41).

As shown in Figure 2E, a 24 h treatment with LAV resulted in the lowest number of bacteria with a log_10_ CFU reduction of 1.65. Nevertheless, PRT (1.43), CA (1.37) and MA (1.36) significantly decreased the number PA within biofilms as well.

**Figure 3:**
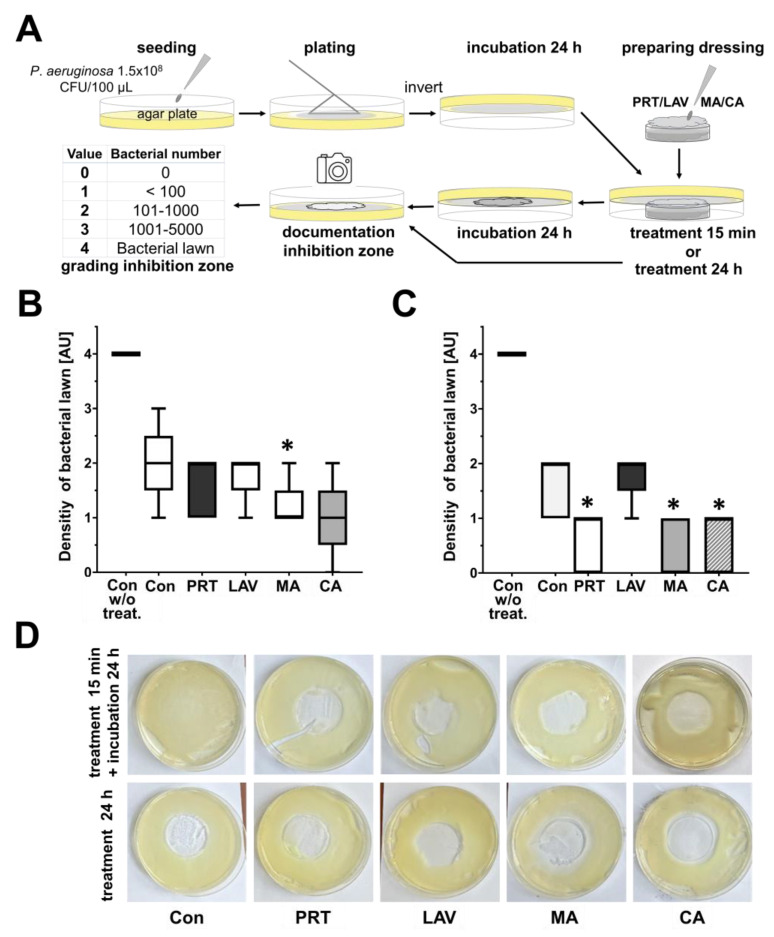
Antibacterial effects of wound dressings soaked in Prontosan, Lavanox, mafenide acetate or citric acid against *P. aeruginosa.* (**A**) The experimental set-up. Box blots with whiskers display the minimum, maximum, median, upper and lower quartiles of values obtained by grading (see table in (**A**)) after (**B**) 15 min and (**C**) 24 h treatments with wound dressing soaked in 0.9% NaCL, (Con), Prontosan (PRT), Lavanox (LAV), mafenide acetate (MA) or citric acid (CA) or without treatment (Con w/o treat.) (*n* = 5, * *p* ≤ 0.05). (**D**) Exemplary agar plates with bacterial lawn and inhibition zones after 15 min treatments followed by 24 h incubation or after 24 h treatment with fully soaked dressing, as indicated.

The effects of dressings soaked with irrigation solutions on bacterial lawns are shown in Figure 3B,C after 15 min or 24 h of treatment. The bacterial lawn after 24 h was fully present in controls (4.0 ± 0). Following 15 min treatments, the density of bacterial lawns was lower in the control using NaCl (2.0 ± 0.7). The maximum reduction in bacteria was achieved with CA (1.0 ± 0.7), followed by MA (1.2 ± 0.4), PRT (1.6 ± 0.5) and LAV (1.8 ± 0.4). By using 24 h treatments, the greatest reductions in bacteria were achieved with MA (0.4 ± 0.5), followed by CA (0.6 ± 0.5), PRT (0.6 ± 0.5) and LAV (1.8 ± 0.4). However, the control using 0.9% NaCl also showed significant inhibitory effects (1.6 ± 0.5). 

### 2.2. Antimicrobial Efficacy of Antiseptic/Wound Irrigation Solutions in an Ex Vivo Skin Wound Model

**Figure 4:**
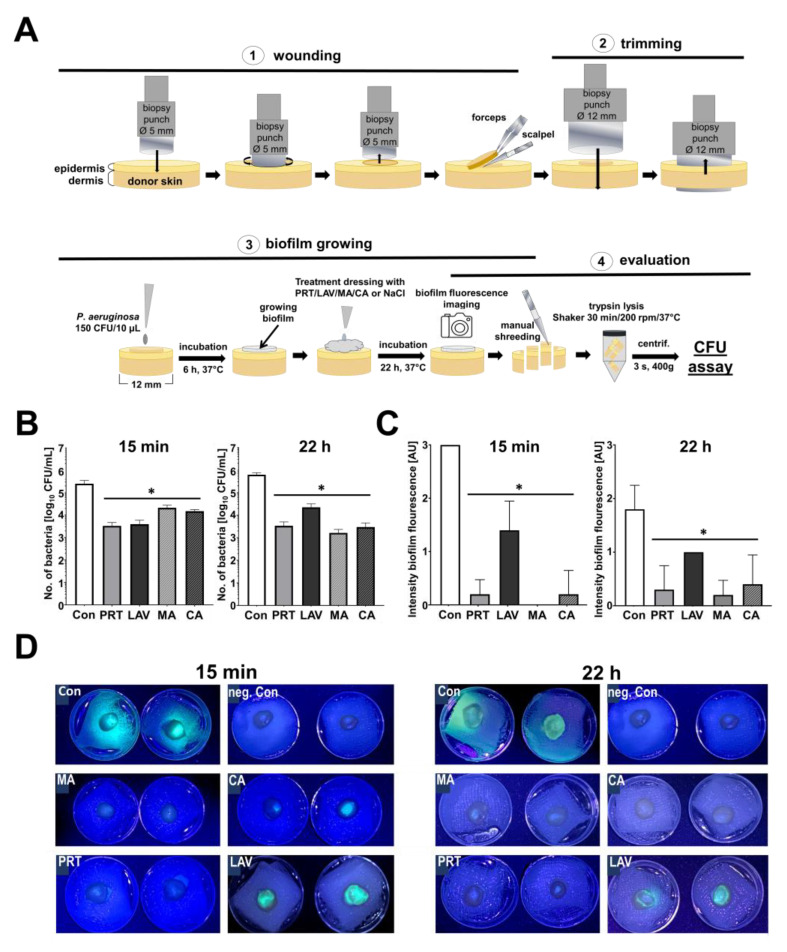
Effects of Prontosan, Lavanox, mafenide acetate or citric acid on *P. aeruginosa*-contaminated wounds ex vivo. (**A**) The experimental workflow. (**B**) Bacterial load (mean + SD; *n* = 5, * *p* ≤ 0.05) in wound skin model after 15 min or 22 h of treatment with Prontosan (PRT), Lavanox (LAV), mafenide acetate (MA) or citric acid (CA). Control (Con) was treated with 0.9% NaCl. (**C**) Qualitative fluorescence signal of contaminated wound skin model after treatments, as indicated (mean + SD; *n* = 5, * *p* ≤ 0.05). Grading: 0 = no fluorescence; 0.5 = very low fluorescence; 1 = low fluorescence; 2 = intermediate fluorescence; 3 = high fluorescence. (**D**) Shown are exemplary photographs of biofilm fluorescence after 15 min/22 h treatments as indicated (neg. Con= negative control with 0.9% NaCl w/o bacteria).

To simulate clinical reality, a wound/skin model had been established earlier [33] (Figure 4A). With this assay, better estimations can be made of the antibacterial effects of the treatments. After 15 min treatments (Figure 4B), the bacterial number on the skin pieces decreased with each treatment in comparison to the control using 0.9% NaCl (5.40 log_10_ CFU/mL). Significantly, the highest reduction was observed on skin samples treated with PRT (3.52 log_10_ CFU/mL), followed by LAV (3.60 log_10_ CFU/mL). 

When treated with CA (4.26 log_10_ CFU/mL) and MA (4.53 log_10_ CFU/mL), the bacterial count was reduced but remained high compared to the other wound irrigation solutions. When the wounds were treated for 22 h (Figure 4B), the bacterial count of the control was 5.79 log_10_ CFU/mL. The highest reduction in bacterial count was achieved after treatment with MA (3.22 log_10_ CFU/mL), followed by CA (3.47 log_10_ CFU/mL), PRT (3.53 log_10_ CFU/mL) and LAV (4.35 log_10_ CFU/mL). Here, the evaluation of biofilm fluorescence showed a clear reduction in fluorescence density with all treatments (Figure 4C,D). The strongest fluorescence signal was produced by the control samples treated with 0.9% NaCl. A decrease in fluorescence signal was observed following treatment with each wound irrigation solution and graded as mentioned in the Methods section. Figure 4C shows that no fluorescence signal was observed on skin samples treated for 15 min with MA (0 ± 0), followed by PRT (0.2 ± 0.3), CA (0.2 ± 0.4) and LAV (1.4 ± 0.5).

All skin samples treated for 24 h had a weaker fluorescence signal than those treated for 15 min. Fluorescence density was the highest when treated with C (1.8 ± 0.4) followed by MA (0.2 ± 0.3), PRT (0.3 ± 0.4), CA (0.4 ± 0.5) and LAV (1.0 ± 0).

## 3. Discussion

In infected burn sites, it is the Gram-negative bacterium *P. aeruginosa* (PA) that is mostly isolated [6,9]. PA’s ability to form a biofilm enables a persistent wound infection and a significant delay in wound healing [9]. Burn wound infections can prolong the hospital stay of patients by up to 9 days or even cause major complications [34,35]. Thus, the main objective in burn wound management is prevention and effective therapy of infected burn wound sites, so that the incidence of major complications such as sepsis and death can be reduced and healing of the damaged skin can be achieved [36]. Consequently, a preliminary daily and specialized wound care regimen is needed. 

The standard wound therapy includes daily wound cleansing and dressing changes. In the burn unit of the Cologne-Merheim hospital, standard wound irrigation solutions are PRT (containing 0.1% polyhexanide and 0.1% betadine) and LAV (containing 0.08% NaOCl/HOCl), both known to have antibacterial effects [28,31].

In our study, both standard wound irrigation solutions, PRT and LAV, were shown to have high antimicrobial efficacy against PA (Figure 1, Figure 2, Figure 3 and Figure 4). In the CFU essay, log_10_ reductions of 4.91 (LAV) and 4.74 l (PRT) in the planktonic PA were shown. With PRT, too, the greatest biofilm reduction was achieved compared to all other wound irrigation solutions after 24 h of treatment in the crystal violet assay. However, whereas PRT, MA and CA showed in the compress assays clear and colony-free inhibitory zones after a 24 h treatment, LAV did not show better results than a treatment with 0.9% NaCl, indicating that the antimicrobial efficacy of LAV was time-limited.

Even more relevant for clinical reality are our results in the wound skin model, showing that PRT and LAV provide the highest bacterial reduction after 15 min of treatment (Figure 4B). Although in vitro experiments are unlikely to mimic in vivo biofilm formation to a full extent, we were able to show in the wound skin model that, after 15 min of treatment, PRT and LAV reduced the bacterial number by 1.8–1.9 log_10_. In addition, biofilm fluorescence was strongly reduced by PRT, MA and CA but not by LAV (Figure 4C,D). Not surprisingly, after the longest treatment period of 24 h, the antibiotic topical treatments with MA showed a greater reduction (~3.6 log_10_ reduction) in the bacterial count than PRT and CA (both ~2.3 log_10_ reduction) (Figure 4B). LAV was shown to have the lowest antimicrobial efficacy and the highest fluorescence signal, both after 15 min and 24 h of treatment. This underlines the known problem of a rapid loss of efficacy of solutions containing sodium hypochlorite (SHC) [28], which may necessitate a more frequent change of LAV dressings in clinic to ensure a stable antibacterial effect. The manufacturers recommend an exposure time of approximately 15 min. In an in vitro study of wound irrigation solutions containing SHC, LAV showed a relevant antimicrobial effect on PA of the six investigated solutions, while also showing a cytotoxic effect on keratinocytes and fibroblasts, depending on the treatment duration and concentration [28]. These cytotoxic effects are why LAV is not recommended to be used on skin grafts [37].

Although in our results, PRT is shown to have a high antimicrobial efficacy; studies have shown that polyhexanide has a lesser effect on PA than on other Gram-negative bacteria [38]. In burn wound care, a daily PRT application and dressing change might lead to the generation of polyhexanide-resistant strains, as already observed with strains of methicillin-resistant staphylococcus aureus [30]. We suggest that further studies with PA isolated from burn wounds should be performed to investigate and demonstrate the susceptibilities of the potentially resistant PA or other microorganisms.

Systemic antimicrobial therapy is not ideal for the local wound therapy of infected burn wound sites. Because wound areas are poorly vascularized, high dosages would be needed, while sources in the literature have proven that systemic administration does not prevent bacterial colonization in burn wounds [36]. A local antibiotic treatment with CA with a dosage of 2% is an alternative and effective therapy option [39]. Similar to our results, it has been shown in the literature that, with CA, bacterial colonisation can be efficiently removed from burn wounds with an additional stimulation of granulation tissue after the seventh treatment. A further advantage of CA is that it does not pose any toxicity towards fibroblasts [19,39]. Another well-known topical therapy option is MA, a broad-spectrum antibiotic and standard therapy for PA-infected burn wounds. MA is also known to provide effective penetration of third-degree burn wound eschar [40].

As expected, our results show that the full antibacterial effects of the antibiotic agents are present after 22 h (Figure 4B). While the highest antibacterial efficacy was shown for CA and MA in the CFU and compress assay, biofilm reduction was significantly lower than LAV and just slightly lower than PRT (Figure 2C). In contrast to our results, in an in vitro study conducted on bacterial isolates from burn wounds, it was shown that MA had no effects on Gram-negative bacterial isolates. This might have been the result of its heavy use in burn units, fostering the generation of potentially resistant organisms [37]. Thus, the findings of our study remain limited, as PA isolated from wounds can exhibit specific resistances, which in turn can vary their susceptibility against PRT, CA, LAV and MA. Here, further studies concerning the antimicrobial efficacy of wound irrigation solutions and topical antibiotics on PA isolated from burn wounds are necessary.

Nevertheless, in order to prevent long-term use and, therefore, the development of resistance, an alternating use of PRT, LAV and possible CA for infection prophylaxis would be conceivable, whereas a combined alternating treatment with CA and MA when wound infections with PA occur can be advocated here. In this way, the pace of resistance-building could be slowed down. 

We are currently handling burn wounds in a similar way in the Clinic for Plastic Surgery at the Cologne-Merheim Hospital. All four solutions are commonly used. PRT is in standard use as a wound irrigation solution and for mechanical cleansing, as well as for moistening and normal wound dressing changes. With the first indications of wound infection, LAV is used in the treatment regime. With a manifested PA infection, MA and CA are alternately used, before radical debridement is carried out, if necessary.

In conclusion, we can, therefore, confirm the antimicrobial efficacy of the antibiotic wound irrigation solutions CA and MA in PA infections under in vitro conditions. The wound irrigation solutions PRT and LAV also show high antimicrobial efficacy in vitro and could also be used therapeutically in prophylaxis and treatment of PA infections. Here, slight advantages are shown for PRT. The alternating or combined use of LAV and PRT could also be interesting and requires further experimental and clinical investigation. 

## 4. Materials and Methods

### 4.1. Wound Irrigation Solutions

In this study, we used Prontosan*^®^* (PRT; B. Braun, Melsungen, Germany), a combination of water, polyaminopropyl biguanide (polyhexanide 0.1%), and betadine (0.1%); Lavanox*^®^* (LAV; Serag Wiessner GmbH & Co KG, Naila, Germany), an oxidative, halogenated, activated mineral salt solution containing 0.08% sodium hypochlorite (NaOCl); citric acid (2%) solution (CA; Central pharmacy of the City Cologne gGmbH); and mafenide acetate (5%) solution (MA; Central pharmacy of the City Cologne gGmbH). Physiological saline solution (NaCl 0.9%) (B. Braun, Melsungen, Germany) was used as a control (C). 

### 4.2. Bacterial strains

*Pseudomonas aeruginosa* (PA) was provided by the Leibniz Institute DSMZ—German Collection of Microorganisms and Cell Culture (DSM 939; batch No.: 0411). The PA subculture II was utilized for the experiments. For a masterplate, a cryoconserved PA sample was grown on a tryptone soya agar (TSA) plate (Sigma-Aldrich, Munich, Germany) for 24 h, which was then stored at 7 °C for up to 2 weeks. One day before the experiments, a colony was picked from the masterplate and incubated in a 25 mL tryptone soya broth (TSB) culture medium at 37 °C on a shaker (200 rpm; CO_2_-resistant, 3 mm Orbit, Thermo Scientific Fisher, Waltham, MA, USA). The obtained PA solution was diluted to 1.5 × 10^8^ CFU/mL using a photometer (Epoch II, BioTek, Winooski, VT, USA) measuring the absorbance at 600 nm, equal to a 0.5 McFarland standard (OD 0.1), and then further diluted in TSB to the exact concentration required for the experiments described below.

### 4.3. Antimicrobial Efficacy of CFU Assay

Antibacterial efficacy of the examined solutions was tested via a quantitative suspension method based on DIN EN 13727 [41]. Therefore, 100 µL bacterial suspensions of 1.5 × 10^6^ CFU *P. aeruginosa* were diluted and incubated with 900 µL of the wound irrigation solutions (Figure 1A) for 15 min at 37 °C. After treatment, dilution sample series and an untreated control were prepared with TSB and dispersed on TSA plates to quantify surviving bacteria. After an overnight incubation (37 °C) in an incubator (Thermo Fisher Scientific, Marietta, GA, USA), the bacterial survival (CFU/mL) and log_10_ CFU reduction were determined.

### 4.4. Biofilm Assay

As shown in Figure 2A, PA cultures (TSB,25 mL) were adjusted to 1.5 × 10^8^ CFU/mL as described above and distributed (200 µL/well) in sterile 96-well plates (Sarstedt, Nümbrecht, Germany) and then cultured in an overnight incubation on a shaker (200 rpm; CO_2_-resistant 3 mm Orbit, Thermo Scientific Fisher, Waltham, MA, USA) placed in an incubator (Hera Cell 240 Incubator, Heraeus, Hanau, Germany, Thermo Fisher Scientific, Marietta, GA, USA) for 24 h at 37 °C. After the incubation, the plates were rinsed five times with PBS to eliminate any remaining planktonic bacteria. The PBS was shaken out by tapping the 96-well plates upside down on a dry paper towel and incubated (15 min, 24 h) with PRT, LAV, CA, MA or C (200 µL). After incubation, the wells were washed twice with PBS, shaken out on a paper towel and dried for 1 h, and then stained with 250 µL of a 0.1% crystal violet solution (Sigma, Deisenhofen, Germany) for 15 min at room temperature. After further washing steps, the biofilm formation was photographed before 250 µL of 96% ethanol/1% hydrochloric acid (1 M) was applied to each well to dissolve the crystal violet. After ten minutes of incubation, 2 × 100 µL from each well was transferred to individual wells of a clean 96-well plate, and the optical densities of the samples at 600 nm were determined using the microplate reader (Epoch 2, BioTek, Winooski, VT, USA). For the calculation, the mean of bacteria-free controls was subtracted. To determine the bacterial load in the cultivated and treated biofilm, parallel to the crystal violet assay, additional 96-well plates containing bacteria were identically created and treated. Here, after the washing steps, instead of crystal violet staining, 250 μL of a freshly produced 10% (*w*/*v*) bromelain solution (Bromelain-POS^®^, RSAPHARM Arzneimittel GmbH, Saarbrücken, Germany) was added to each well and incubated for 1 h at 37 °C on a shaker (150 rpm). Bromelain, as shown in preliminary investigations, is capable of dissolving biofilms without displaying considerable antibacterial effects [42]. Then, the bromelain supernatant from three identically treated wells was pooled and directly used for CFU assays.

### 4.5. Compress Assay

To determine the antimicrobial efficacy and obtain qualitative data on the various wound irrigation solutions, sterile gauze pads (ES-Gauzes, 10 × 10 cm, Hartmann, Germany) were inoculated on PA-contaminated agar plates (Figure 2). One day before the treatment with wound irrigation solutions, 100 µL of bacterial solution (adjusted to 1.5 × 10^8^ CFU/mL) was plated out on TSA plates. After incubation (24 h), sterile gauzes, cut into 2 × 2 cm pieces, were separately immersed in the wound irrigation solutions (PRT, LAV, CA, MA or C). Each soaked gauze piece was placed in the centre of one of the prepared agar plates with fully-grown bacterial lawns for 15 min or 24 h and removed afterwards. After a 15 min treatment, the agar plates were also incubated for 24 h to determine the effects on the bacterial lawns by estimating the number of colonies inside the treatment area (Figure 3A).

### 4.6. Wound Skin Model

The experimental procedure is shown in Figure 4. Human donor skin specimens were obtained from abdominoplasty surgeries conducted at the Clinic for Plastic Surgery, Hand Surgery, and Burn Centre at the Cologne-Merheim Hospital. The use of human skin was approved by the ethics committee of the University of Witten/Ethics Herdecke’s Committee (Votum No. 15/2018), and all experiments were conducted with patients’ consent in compliance with the Declaration of Helsinki Principles.

Skin specimens donated by five female patients aged between 27 and 49 years (mean age 42 years) were used for the experiments. All skin specimens were transported postoperatively in a sterile container on ice to the laboratory. The cleansing procedure contained one washing step for one minute with 70% ethanol (Carl Roth, Karlsruhe, Germany) before further processing. First, wound areas were prepared with a biopsy punch (5 mm; Acuderm Inc., Fort Lauderdale, FL, USA). Here, an area of the skin was punched to a depth of ~1 mm, and then the epidermis was removed in this area with the help of forceps and a scalpel (BRAUN/Aesculap AG, Tuttlingen, Germany). Secondly, with a bigger biopsy punch (12 mm; Acuderm Inc., Fort Lauderdale, FL, USA), round skin samples were punched around the 5 mm wounds. Theses skin samples were directly placed in 6-well culture plates onto sterile gauze pads (1.5 × 1.5 cm) filled with 5.5 mL of supplemented cell culture medium (DMEM w/o phenol red; 1.0 g/L glucose, with 10% foetal calf serum, PAN Biotech, Aidenbach, Germany). Finally, a volume of 3.3 μL TSB containing PA (50 CFU) was applied onto each wound area, and the skin/wound samples were incubated for 6 h at 37 °C. 

After incubation, PRT, LAV, MA, CA and C were applied onto small pieces of sterile gauze compress pads and were placed on the wound areas and incubated for 15 min or 22 h (37 °C). The gauze pads were removed after the 15 min incubation, and the skin/wound samples were then incubated for 22 h at 37 °C. All treatments were performed in duplicates. After incubation, duplicates were pooled and cut into small pieces, then transferred in centrifugation tubes and enzymatically digested in 5 mL of 0.25% trypsin/HBSS solution (PAN Biotech, Aidenbach, Germany) in the incubator on a shaker for 30 min (150 rpm). After a short centrifugation (3 s), 100 μl the respective solutions was placed on agar plates to determine the bacterial survival rate (CFU/mL).

The biofilms produced by PA are known to produce fluorescence-showing pigments in the violet spectra [43]. Therefore, prior to the cutting/enzymatic digestion, we examined the bacterial biofilm fluorescence on the skin/wound samples with a commercially available LED black-light torch (395 nm, Bestsun, Jiaxing, China) and took photos in a darkroom with a fixed exposure time. The biofilm fluorescence intensities were evaluated 22 h after treatment and graded by two independent examiners on a scale of zero to three compared to the specific untreated control denoting the following signal intensities: 0 = no fluorescence; 0.5 = very low fluorescence; 1 = low fluorescence; 2 = intermediate fluorescence; and 3 = high fluorescence.

### 4.7. Statistics

GraphPad Prism Version 8.4.3 (San Diego, CA, USA) was used for statistical analyses. Significant differences were evaluated using one-way ANOVA. A *p*-value of ≤0.05 was considered significant. The Kruskal–Wallis test, as a nonparametric test, was used for the wound compress assay and biofilm fluorescence analysis.

## Data Availability

The data that support the findings of this study are available from the corresponding author upon reasonable request.
